# The uptake and excretion of partially oxidized sulfur expands the repertoire of energy resources metabolized by hydrothermal vent symbioses

**DOI:** 10.1098/rspb.2014.2811

**Published:** 2015-05-07

**Authors:** R. A. Beinart, A. Gartman, J. G. Sanders, G. W. Luther, P. R. Girguis

**Affiliations:** 1 Department of Organismic and Evolutionary Biology, Harvard University, Cambridge, MA 02136, USA; 2 School of Marine Science and Policy, College of Earth, Ocean and Environment, University of Delaware, Lewes, MD, USA

**Keywords:** symbiosis, hydrothermal vent, sulfur metabolism, ecosystem engineer

## Abstract

Symbiotic associations between animals and chemoautotrophic bacteria crowd around hydrothermal vents. In these associations, symbiotic bacteria use chemical reductants from venting fluid for the energy to support autotrophy, providing primary nutrition for the host. At vents along the Eastern Lau Spreading Center, the partially oxidized sulfur compounds (POSCs) thiosulfate and polysulfide have been detected in and around animal communities but away from venting fluid. The use of POSCs for autotrophy, as an alternative to the chemical substrates in venting fluid, could mitigate competition in these communities. To determine whether ESLC symbioses could use thiosulfate to support carbon fixation or produce POSCs during sulfide oxidation, we used high-pressure, flow-through incubations to assess the productivity of three symbiotic mollusc genera—the snails *Alviniconcha* spp. and *Ifremeria nautilei*, and the mussel *Bathymodiolus brevior*—when oxidizing sulfide and thiosulfate. Via the incorporation of isotopically labelled inorganic carbon, we found that the symbionts of all three genera supported autotrophy while oxidizing both sulfide and thiosulfate, though at different rates. Additionally, by concurrently measuring their effect on sulfur compounds in the aquaria with voltammetric microelectrodes, we showed that these symbioses excreted POSCs under highly sulfidic conditions, illustrating that these symbioses could represent a source for POSCs in their habitat. Furthermore, we revealed spatial disparity in the rates of carbon fixation among the animals in our incubations, which might have implications for the variability of productivity *in situ*. Together, these results re-shape our thinking about sulfur cycling and productivity by vent symbioses, demonstrating that thiosulfate may be an ecologically important energy source for vent symbioses and that they also likely impact the local geochemical regime through the excretion of POSCs.

## 1. Introduction

Hydrothermal vents support dense assemblages of invertebrates, many of which rely on chemoautotrophic bacterial symbionts for nourishment. These microbial–animal associations aggregate around vent orifices so that their symbionts can use chemicals in the venting fluid for the energy to support carbon fixation [1]. The symbionts of animals from multiple phyla have been shown to use sulfide, methane and/or hydrogen from vent fluid as energy sources [2–11]. Though these are important substrates for microbial energy production [12], other reduced compounds can be present in and around venting fluid and, to date, less is known about the extent to which vent symbioses use these compounds [13–16]. Some of these, including the partially oxidized sulfur compounds (POSCs), polysulfide and thiosulfate, are produced from the abiotic and/or biological oxidation of vent-derived sulfide [13,17]. POSCs are especially abundant away from hotter venting fluids, where the concentrations of sulfide and other reductants sourced directly from the fluid have become depleted or diluted [13,17–19]. As competition among vent symbioses for vent-derived resources (manifest as competition for space around a vent) is likely to be intense, POSCs could be ecologically advantageous for organisms that can use these compounds, as it could lessen the competition for space around vigorous venting. Additionally, use of POSCs could mitigate exposure to the high temperatures and toxic chemicals (e.g. heavy metals) that accompany the chemical reductants in venting fluid, providing additional advantage for the hosts, which like all animals, exhibit some sensitivity to such conditions.

It is well known that chemoautotroph–animal symbioses from non-vent ecosystems can use POSCs for energy generation [20–23]. Moreover, some free-living, vent chemoautotrophs can use POSCs [24,25], and thiosulfate-driven autotrophy has been observed among the epibiotic symbionts of vent crustaceans [6–8,26]. While a few studies have measured thiosulfate oxidation *ex hospite* by the intracellular symbionts of vent mussels and clams [2,5,9,27,28], thiosulfate-driven autotrophy has not been demonstrated in an intact vent symbiosis, and the extent to which thiosulfate and other POSCs support productivity is unknown.

The distribution and abundance of POSCs in vent fields has been best characterized along the Eastern Lau Spreading Center (ELSC), which is in the southwestern Pacific near the island of Tonga. Symbiotic mollusc species from three genera dominate at these vent fields: three cryptic provannid snail species from the genus *Alviniconcha*, another Provannid snail *I. nautilei* and the mussel *B. brevior* [29]. Each species associates with different, phylogenetically distant lineages of symbiotic, intracellular bacteria [30–38] that are capable of supporting autotrophy using sulfide [39]. Typically, these animals form concentric patterns around vent orifices, with *Alviniconcha* spp*.* found closest to the vent, followed by a zone of *I. nautilei*, and finally *B. brevior* at the very edges of the assemblage [18,40,41]. It has been suggested that these zones form as a result of competitive interactions for vent-derived resources as well as due to differences in temperature and chemical tolerance or preference [41,42]. Thiosulfate and polysulfides (POSCs) are common in and around these concentric aggregations at concentrations up to 1000 and 400 µM, respectively, which are equal to or greater than the concentrations of sulfide and likely other reductants from venting fluid in the same areas [13,17,18]. Strikingly, the diversity and abundance of POSCs correspond to the distribution of the mollusc genera. In particular, the highest concentrations of thiosulfate in the aggregations are found over the zones of *B. brevior* [17,18], while the highest concentrations of polysulfides are found among *Alviniconcha* spp. and *I. nautilei* [13,18].

To determine whether these symbioses could use POSCs to support carbon fixation or produce them during sulfide oxidation, we conducted a series of shipboard incubations with all three ELSC mollusc symbioses using high-pressure, flow-through aquaria. Inline voltammetric electrodes allowed us to assess total sulfur flux through the aquaria, while the use of isotopically labelled inorganic carbon allowed us to quantify individual productivity during the incubations.

## 2. Material and methods

### (a) Animal collections

Animals were collected from the vent fields ABE (−20°45.8′ by −176°11.5′) or Tu'i Malila (−21°59.4′ by −176°34.1′) at the ELSC by the remotely operated vehicle *JASON II* during the R/V *Thomas G. Thompson* expedition TM-235 in 2009. *Alviniconcha* spp*.*, *I. nautilei* and *B. brevior* were recovered in insulated containers and, once on board, were briefly kept in 4°C seawater. Individuals responsive to touch were placed in the flow-through titanium aquaria of the high-pressure respirometry system (HPRS; electronic supplementary material, Methods and figure S1; [4,43]). For each incubation, between three and 10 individuals of each genus were placed into three separate aquaria. Animals were situated upon perforated acrylic partitions so that they were stacked vertically in the cylindrical aquaria.

### (b) Incubation conditions and acclimation

Three incubations (hereafter ‘rate experiments’) were performed to compare net sulfur uptake and excretion rates, as well as carbon fixation rates, by the three mollusc genera at three different conditions: 105 µM sulfide, 300 µM thiosulfate and sulfur-free. During each rate experiment, an empty high-pressure aquarium (control) was run alongside the three animal-containing aquaria in order to account for systematic losses and enable the most robust mass-specific rate determinations. Two additional incubations (hereafter ‘variation experiments’) were performed to establish the extent of variation in carbon fixation rates among the animals within each aquarium. Variation experiments were run at 350 µM sulfide or 300 µM thiosulfate, up to twice the number of individuals per aquaria than in the rate experiments.

Animals were acclimated to the aquaria by incubating in aerated seawater at 15°C and 25 MPa for 8 h prior to the start of each experiment or treatment. Similarly, during the sulfur-free experiment, animals were acclimated with approximately 300 µM sulfide for 8 h before sulfur was stopped and isotopic tracer was added**.** As the sulfur-free experiment required a prolonged period without exogenous reductants, acclimation with sulfide allowed normalization of physiological states including, potentially, the accumulation of sulfur stores.

### (c) Sulfur oxidation and excretion rates

To determine the net sulfur oxidation rates, as well as to detect the excretion of POSCs, the concentration of sulfur compounds in the aquaria effluent were compared to the effluent from the empty control aquarium (rate experiments) or to the concentrations in input water (variation experiments). The concentrations of total sulfide (∑H_2_S and HS^−^), thiosulfate and polysulfides were measured via voltammetric microelectrodes [15,44]. Though we were unable to quantify oxygen concentrations during the incubations at high concentrations of sulfide, the voltammetric microelectrodes (see below) were able to always detect oxygen in the aquaria effluent during all incubations (minimum detection limit is 5 µM; [13]), suggesting that oxygen was never completely depleted by the symbioses in the aquaria (see electronic supplementary material, Methods).

The input and/or effluent water was measured in a cyclical series of 30 min intervals throughout the duration of the experiment; one complete 2-h series of four intervals starting with an experimental effluent and ending with either the control effluent or input water is hereafter referred to as a ‘set’. At the start of every 15 min period, a series of 21 electrochemical scans were performed at a rate of one per approximately 2 s, resulting in 42 measurements per 30 min interval. All net sulfur oxidation rates were calculated using data obtained after 10 h or more into the experiment, which allowed at least three turnovers in the volume of water in the aquaria. To calculate the concentrations of each sulfur compound during each 30 min interval, the first 10 scans, or 24%, in each interval were removed to exclude the transitory scans that occurred after the switch to a different aquarium effluent. The concentrations resulting from the remaining scans were averaged, resulting in a single concentration value per interval (hereafter, ‘interval concentration’). To calculate mass-specific oxidation rates for each mollusc genus within a set, the interval concentration of the control effluent was subtracted from the interval concentration of the experimental effluent, which in turn was divided by the total gill mass of the animals in each aquarium and multiplied by the fluid flow rate through the aquaria in litres per hour.

### (d) Sampling of experimental animals

At the conclusion of each experiment, aquaria were depressurized and animals were quickly removed, excised from their shells and weighed via a motion-compensated shipboard balance [45]. Each individual's gill weight was estimated from linear equations derived from the regression of total body mass to gill mass (electronic supplementary material, figure S2) for each genus. Gill and foot tissue were frozen at −80°C for isotopic analysis and gill tissue was homogenized and preserved in Trizol™ (Life Technologies) for nucleic acid extraction.

### (e) Symbiont identities

DNA was extracted from the Trizol™-preserved tissue samples following RNA extraction via a modified manufacturer's protocol. Symbiont 16S rRNA genes from *I. nautilei* and *B. brevior* associations were amplified via PCR using universal bacterial primers [46] and directly Sanger sequenced. Because some *Alviniconcha* spp. from the ELSC are known to host mixed assemblages of phylogenetically distinct symbionts [34], direct sequencing of the symbiont populations was not possible for this genus. Thus, symbiont populations associated with the experimental *Alviniconcha* spp. were assessed via qPCR as in Beinart *et al*. [34]. Subsequently, a Bayesian inference phylogeny was produced with MrBayes [47] (see electronic supplementary material, Methods).

### (f) Carbon stable isotopic analyses and carbon fixation rates

DIC isotopic compositions were determined at the Yale Institute of Biospheric Studies Earth System Center for Stable Isotopic Studies, using well-established protocols (see electronic supplementary material, Methods). Approximately 300 mg of symbiont-containing gill and symbiont-free foot tissue were subsampled while frozen for carbon isotopic analysis. Samples were lyophilized for 24 h then acidified with 0.1 N HCl to remove unincorporated Na^13^CO_3_. The samples were subsequently dried for 24–48 h at 50–60°C, weighed to determine dry weight, then homogenized to a fifine powder. The carbon isotopic composition and per cent carbon content were determined for foot and gill tissue samples via isotope ratio mass spectrometry (see electronic supplementary material, Methods).

To obtain the percentage of ^13^C incorporated during these experiments (%^13^C_inc_), the following formula, modified from Riou *et al.* [48] was used
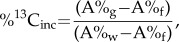


where A%_g_ is the atomic per cent of the symbiont-containing gill tissue sample in which carbon incorporation occurs; A%_f_ is the atomic percent of the same individual's foot tissue sample and A%_w_ is the atomic per cent of the input water DIC containing the added isotopic label. Because foot tissue in these animals is symbiont-free and unlikely to receive appreciable quantities of translocated carbon from the gill over the short time course of these experiments, it serves to represent the initial A% of the individual in the above equation.

The weight of incorporated carbon (W^13^C_inc_) was calculated by multiplying the %^13^C_inc_ by the dry weight of the gill tissue (DW_g_) and the carbon content of the sample (%C). Carbon incorporation rates (C_inc_) are expressed as micromoles ^13^C per gram of wet tissue per hour. This was first calculated as a rate of carbon incorporation expressed as micromoles ^13^C per gram of dry tissue per hour (DryC_inc_) with the formula
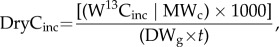
where MW_c_ is the molecular weight of ^13^C; and *t* is the duration of the experiment. DryC_inc_ was then converted to C_inc_ by multiplying dry C_inc_ by the ratio of DW_g_ to the wet weight of that same sample (W_g_).

Additional details may be found in the electronic supplementary material, Methods.

## 3. Results

### (a) Sulfur metabolism and carbon fixation in rate experiments

**Figure 1.**
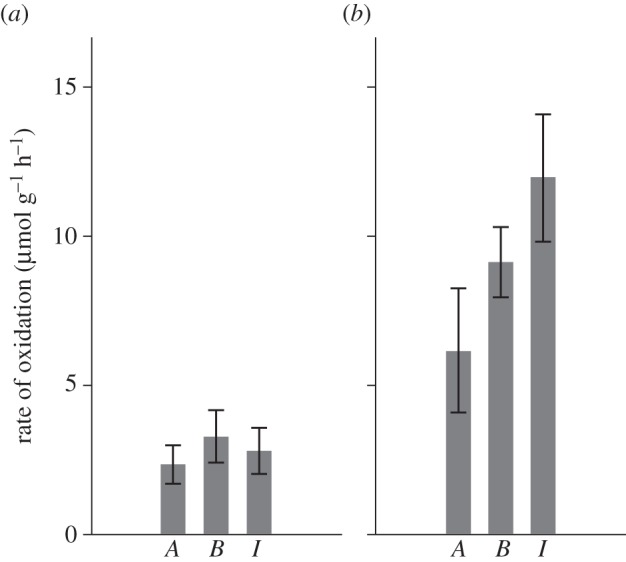
Average mass-specific net sulfur oxidation rates (micromoles of sulfur per gram of wet gill tissue per hour) ±s.d. during the sulfide (*a*) and thiosulfate (*b*) rate experiments for *Alviniconcha* spp. (*A*), *B. brevior* (*B*) and *I. nautilei* (*I*).

All three symbioses demonstrated net sulfide and thiosulfate uptake (figure 1 and table 1). Though sulfur concentrations were diminished by the animals during the rate experiments (relative to the control aquarium), measurable concentrations of added sulfide and thiosulfate were detected in the aquaria effluent, suggesting that sulfur compounds did not become limiting. Net sulfide oxidation rates were comparable among the three genera, though *B. brevior* had the highest average rate at these experimental conditions (figure 1*a*). Net thiosulfate oxidation rates were more varied among the three genera, with *I. nautilei* having almost twice the average rate of *Alviniconcha* spp. (figure 1*b*). Additionally, thiosulfate oxidation rates fluctuated more widely over the duration of the experiment than did sulfide oxidation rates (figure 1*a*,*b*). No additional sulfur compounds were detected in the effluent of the three experiments, indicating that biologically mediated sulfur transformations and excretions did not occur at these conditions.

**Table 1.** Net sulfur uptake (oxidation) and excretion by the three mollusc genera. Number of individuals (*n*) and the total wet weight of gill tissue for each mollusc genus in each experiment, as well as the average (min, max) concentrations of sulfide, thiosulfate and polysulfides as measured via a voltammetric electrode in the effluent from each aquaria. BDL stands for ‘below detection limit’.

experiment	genus	*n*	total gill wet weight (g)	sulfide (μM)^a^	thiosulfate (μM)^a^	polysulfides (μM)^a^
sulfur-free	control	0	0	BDL	BDL	BDL
	*Alviniconcha*	5	15.88	BDL	BDL	BDL
	*I. nautilei*	5	18.83	BDL	BDL	BDL
	*B. brevior*	4	18.78	BDL	BDL	BDL
sulfide	control	0	0	66 (46, 107)	BDL	BDL
	*Alviniconcha*	5	22.56	5.7 (2.7,9.7)	BDL	BDL
	*I. nautilei*	5	20.49	5.8 (3.0, 8.6)	BDL	BDL
	*B. brevior*	4	18.36	3.0 (1.4, 3.9)	BDL	BDL
thiosulfate	control	0	0	0.32 (BDL, 1.1)	276 (216, 310)	BDL
	*Alviniconcha*	3	21.42	0.22 (BDL, 0.36)	139 (BDL, 209)	BDL
	*I. nautilei*	4	19.50	0.22 (BDL, 0.31)	42 (BDL, 70)	BDL
	*B. brevior*	3	13.46	BDL	140 (67 188)	BDL

^a^Detection limits: sulfide and polysulfides, 0.20 μM; thiosulfate, 30 μM.

**Figure 2.**
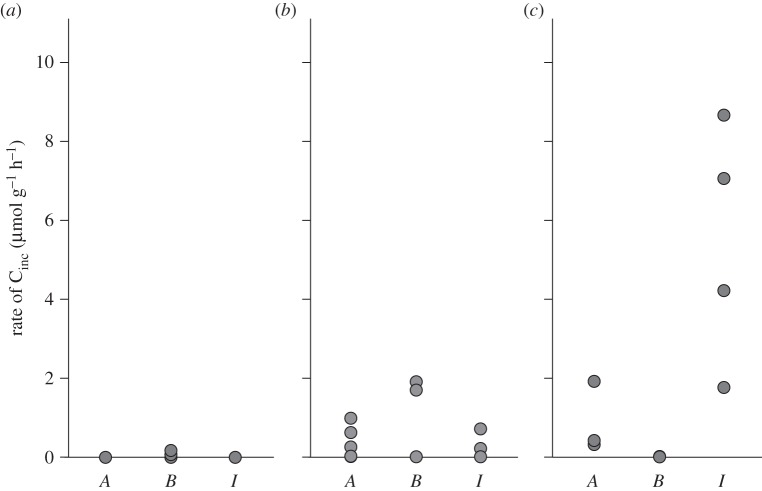
Individual mass-specific carbon incorporation rates (micromoles of ^13^C per gram of wet gill tissue per hour) for *Alviniconcha* spp. (*A*), *I. nautilei* (*I*) and *B. brevior* (*B*) during the (*a*) sulfur-free, (*b*) sulfide and (*c*) thiosulfate rate experiments.

Carbon incorporation (fixation) was stimulated in the gills of individuals from all three symbiotic genera when sulfide was provided (figure 2*b*; electronic supplementary material, table S1). By contrast, only *Alviniconcha* spp. and *I. nautilei* individuals fixed carbon when given thiosulfate (figure 2*c*). Carbon incorporation rates among the genera did not differ significantly from one another during the sulfide experiment (Kruskal–Wallis, *p* = 0.424) (figure 2*b*). Among all three experiments, the greatest rates of carbon fixation occurred in *I. nautilei* individuals supplied thiosulfate (figure 2*c*), though this was not significantly different from *Alviniconcha* spp. individuals in that experiment (Mann–Whitney *U*, *p* = 0.114; *B. brevior* was omitted from this comparison as no carbon incorporation was observed in the thiosulfate treatment). Carbon fixation was not stimulated in *Alviniconcha* spp. and *I. nautilei* individuals in the sulfur-free (control) experiment, though minor carbon incorporation was detected in two of the four *B. brevior* individuals.

### (b) Sulfur metabolism and carbon fixation during variation experiments

During the variation experiments, sulfide and thiosulfate concentrations were depleted in the effluents of the aquaria in their respective treatments (relative to the input water; table 2). As with the rate experiments, measurable concentrations of added sulfide and thiosulfate were detected in the experimental effluent of all incubations, indicating that the symbioses did not completely exhaust these compounds in the aquaria. Mass-specific uptake (oxidation) rates were comparable among treatments, though the average sulfide oxidation rate was greater than the average thiosulfate oxidation rate for all three genera (table 2). Sustained sulfur excretion was observed throughout the duration of the sulfide treatment, but not during the thiosulfate treatment (table 2). When exposed to sulfide, both *Alviniconcha* spp. and *B. brevior* released thiosulfate, at rates equivalent to 24–30% and 5–5.2% of their sulfide oxidation rates, respectively. *Ifremeria nautilei* released polysulfide at rates equivalent to 11–47% of its rate of sulfide oxidation.

**Table 2.** Net sulfur uptake (oxidation) and excretion by the three mollusc genera during variation experiments. Number of individuals (*n*); the sum total wet weight of gill tissue for each mollusc genus; average (min, max) concentrations of sulfide, thiosulfate and polysulfides as measured via a voltammetric electrode in the effluent from each aquaria; average (±s.d.) rates of net oxidation or excretion (indicated as negative or positive values, respectively) as micromoles per gram of wet gill tissue per hour.

genus	*N*	total gill wet weight (g)		sulfide^a^	thiosulfate^a^	polysulfides^a^
sulfide treatment						
input	n.a.	n.a.	concentration (μM)	349 (329, 387)	BDL	BDL
*Alviniconcha*	10	36.85	concentration (μM)	21 (19, 23)	73 (68, 77)	BDL
			rate (μmoles g^−1^ h^−1^)	−6.8 ± 0.50	+0.92 ± 0.05	BDL
*I. nautilei*	5	34.08	concentration (μM)	85 (76, 90)	BDL	78 (0.2, 126)
			rate (μmoles g^−1^ h^−1^)	−7.2 ± 0.75	BDL	+2.2 ± 1.5
*B. brevior*	6	39.87	concentration (μM)	14 (12, 15)	34 (30, 40)	BDL
			rate (μmoles g^−1^ h^−1^)	−7.4 ± 0.52	+0.10 ± 0.08	BDL
thiosulfate treatment						
input	n.a.	n.a.	concentration (μM)	BDL	302 (251, 404)	BDL
*Alviniconcha*	8	39.29	concentration (μM)	BDL	40 (30, 82)	BDL
			rate (μmol g^−1^ h^−1^)	BDL	−5.62 ± 1.22	BDL
*I. nautilei*	10	50.68	concentration (μM)	BDL	44 (30, 60)	BDL
			rate (μmol g^−1^ h^−1^)	BDL	−4.66 ± 0.93	BDL
*B. brevior*	6	45.29	concentration (µM)	BDL	103 (30, 162)	BDL
			rate (µmol g^−1^ h^−1^)	BDL	−4.02 ± 0.84	BDL

^a^Detection limits: sulfide and polysulfides, 0.20 µM; thiosulfate, 30 µM.

**Figure 3.**
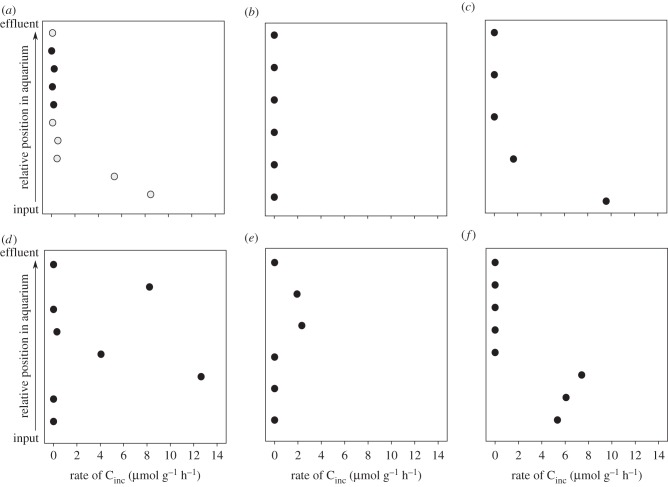
Individual mass-specific carbon incorporation rates (micromoles of ^13^C per gram of wet gill tissue per hour) during the variation experiments. Sulfide variation experiment with (*a*) *Alviniconcha* spp., (*b*) *B. brevior* and (*c*) *I. nautilei*; thiosulfate variation experiment with (*d*) *Alviniconcha* spp., (*e*) *B. brevior* and (*f*) *I. nautilei*. In panel (*a*), grey symbols indicate *Alviniconcha* spp. hosting *ε*-proteobacterial symbionts; black symbols indicate *Alviniconcha* spp. hosting *γ*-1 symbionts. All individuals are shown according to their relative position in the HPRS aquaria.

*Alviniconcha* spp. and *I. nautilei* individuals from both the sulfide and thiosulfate treatments incorporated carbon (figure 3*a*,*c*,*d*,*f*; electronic supplementary material, table S1). No carbon incorporation was observed by *B. brevior* in the sulfide treatment (figure 3*b*), though during the thiosulfate treatment two of the six *B. brevior* individuals incorporated carbon (figure 3*e*). Within both the sulfide and thiosulfate treatments, carbon incorporation rates were greatly variable among individuals. Further examination of these data suggests that the observed variability is likely related to substrate depletion (e.g. sulfur or oxygen) in the aquaria. Specifically, animals positioned in the aquaria closest to the input of water had the highest carbon fixation rates (figure 3). Additionally, individuals from these experiments demonstrated the highest rates of carbon fixation among all of the animals in this study. *Alviniconcha* spp. and *B. brevior* individuals from the thiosulfate variation experiment showed the highest rates of carbon fixation among all individuals of their genus in this study. Additionally, an individual from the sulfide variation experiment had the highest carbon fixation rate for any experimental *I. nautilei*.

### (c) Symbiont identity

Assessment of the symbiont populations through analysis of the 16S rRNA genes demonstrated that the symbionts of each host genus associated with *γ*-proteobacterial symbionts. As indicated by their 16S rRNA genes, there was low diversity among the symbionts of *I. nautilei* and *B. brevior*. Amplification and direct sequencing resulted in clean chromatograms with no evidence of mixed symbiont populations within individuals. Individuals of *I. nautilei* yielded six unique sequences that were at least 99.5% identical to one another, whereas *B. brevior* individuals yielded three unique sequences that were at least 98.8% identical to one another. Bayesian phylogenetic analysis (electronic supplementary material, figure S3) showed that the experimental *B. brevior* symbiont sequences fell in a well-supported clade consisting of symbionts from *Bathymodiolus* mussels and *Calyptogena* clams and a few free-living marine bacteria. Experimental *I. nautilei* symbiont sequences fell in a well-supported clade of *I. nautilei* symbionts from the Lau basin and other hydrothermal vent regions.

With the exception of some individuals in the sulfide variation experiment, all experimental *Alviniconcha* spp. were dominated by symbionts from one of the two *γ*-proteobacterial phylotypes (i.e. more than or equal to 93% of the detected 16S rRNA genes; electronic supplementary material, table S2) that associate with this genus in the ELSC. Most of the individuals in the rate experiments and thiosulfate variation experiment hosted mainly *γ*-1 symbionts; only two *Alviniconcha* from the sulfur-free experiment, which showed no measurable sulfur metabolism or carbon incorporation, were dominated by *γ*-Lau symbionts (*sensu* [34]). Thus, most of the results regarding *Alviniconcha* metabolism herein are in regards to *Alviniconcha* spp. hosting *γ*-1 symbionts. However, six of the 10 *Alviniconcha* in the sulfide variation experiment hosted *ε*-proteobacterial symbionts, and, accordingly, the rates of sulfide oxidation and thiosulfate excretion in that experiment reflect the net effect of both *ε*-proteobacterial and *γ*-1 symbionts’ metabolic activity.

## 4. Discussion

### (a) The significance of thiosulfate oxidation for hydrothermal vent symbioses

Here, we demonstrated, for the first time *in hospite*, that exogenous thiosulfate drove autotrophy in animal–microbial hydrothermal vent symbioses. Two previous *in vitro* and *ex hospite* studies have shown that the intracellular symbionts of *Bathymodiolus* mussels and *Calyptogena* clams support autotrophy via thiosulfate [2,5,9,27,28]. However, as many animals produce thiosulfate from sulfide as a detoxification mechanism [49], these experiments left it ambiguous whether the symbionts were only able to use endogenous thiosulfate produced by their host, or if the intact symbioses could take up and use thiosulfate from their surroundings. The use of stable isotopically labelled inorganic carbon in our experiment revealed that three mollusc symbioses were able to support autotrophy through the uptake of exogenous thiosulfate.

Sulfide oxidation is often considered the main driver of primary productivity by chemoautotrophs at vent habitats [12]; however, the complete oxidation of thiosulfate with oxygen is comparable in energy output to the complete oxidation of HS^−^ (standard Gibbs free energies of −738.7 and −732.6 kJ mol substrate^−1^, respectively [50]). In our experiments, individual thiosulfate-dependent carbon fixation rates often met or exceeded individual carbon fixation rates at a comparable sulfide concentration. Thiosulfate is non-toxic and is more stable with respect to abiotic oxidation than sulfide [51]. As a result, it may be readily concentrated within the host's or symbionts’ cells, leading to a high favourability of reaction resulting from an increased abundance of the substrate. Thus, thiosulfate has the potential to be an important energy source for some hydrothermal vent symbioses, though the extent to which the ELSC symbioses (or others) preferentially use thiosulfate remains to be determined.

Our sulfur-free experiment further emphasizes the value of using thiosulfate as a reductant in the highly variable vent environment. It has long been hypothesized that symbionts could ‘buffer’ the variability in sulfide that arises from variations in flow by using stored, intracellular elemental sulfur granules when exogenous reductants are absent [38,52]. However, for these symbioses, the absence of exogenous sulfur compounds resulted in a cessation of autotrophy (note that all animals were exposed to sulfide during the acclimation period, and thus had the opportunity to build up sulfur stores). For these symbioses, flexible use of multiple sulfur compounds, rather than use of stored compounds, may help them contend with the dynamic conditions at hydrothermal vents. Given the variability in exposure to vent fluid that symbioses likely experience due to temporal and spatial fluid dynamics, the ability to use multiple reductants (e.g. both sulfide and thiosulfate) may relieve the energy limitation that could occur if a symbiosis was exclusively dependent on energy sources found only in venting fluid.

### (b) Sulfur oxidation by the mollusc symbioses at the Eastern Lau Spreading Center

It has been suggested that thiosulfate may be an especially important energy source for *B. brevior*, as it may enable exploitation of habitat away from the venting fluid, where conditions are tolerable but energetic resources derived directly from venting fluid are scarce [18]. Like in previous observations [39], we saw that sulfide-driven carbon incorporation by *B. brevior* only occurred only in the presence of lower sulfide concentrations, but not with the relatively high concentrations of sulfide in the variation experiment. This bolsters the hypothesis that physiological intolerance to the high concentrations of sulfide associated with exposure to venting fluid prevents *B. brevior* from inhabiting areas near vent sources. However, while we observed that *B. brevior* can indeed use thiosulfate to power autotrophy, only two of the nine total individuals tested exhibited carbon incorporation in the presence of thiosulfate (figures 2 and 3), suggesting an inefficient coupling between the metabolic processes. While previous studies have shown that non-vent bivalves with chemoautotrophic symbionts can support autotrophy with thiosulfate [20,21,23], the ability of *B. brevior* symbionts to support autotrophy with thiosulfate *in situ* remains to be determined.

The snails *Alviniconcha* spp. and *I. nautilei* typically live in close proximity to the venting source, where sulfide is more abundant and thiosulfate concentrations are low [13,18,41]. This correlation has led to the suggestion that sulfide is the main energy source for these snails, while thiosulfate is either not being used or is not important. Our results were congruent with a previous report of autotrophy powered by sulfide oxidation in these taxa [39], though our *I. nautilei* mass-specific carbon fixation rates were higher. As rates reported by Henry *et al*. [39] were based on the net uptake of carbon dioxide, high rates of respiration by *I. nautilei* may have masked higher rates of carbon fixation in their experiments. Our isotopic labelling experiments suggest that *I. nautilei* may be as productive as *Alviniconcha* spp. at our experimental conditions (i.e. when sulfide and oxygen are replete). We also observed robust coupling between thiosulfate oxidation and carbon fixation in *I. nautilei* and *Alviniconcha* spp. Consequently, the observation that *I. nautilei* and *Alviniconcha* spp. commonly inhabit regions of low thiosulfate may simply reflect their influence on the pool of thiosulfate. This supposition is supported by the observed increase in thiosulfate concentrations when *Alviniconcha* spp. and *I. nautilei* were removed from snail and mussel aggregations *in situ* [17].

As both sulfide and thiosulfate drove carbon incorporation by all three symbioses, binary differences in the ability to use these compounds do not appear to drive the observed differences in habitat utilization. However, the relative significance of these energy sources to each symbiotic taxon, their contribution to the overall productivity of the community and comparisons of sulfur metabolism among *Alviniconcha* spp. hosting different symbiont phylotypes will require further work.

### (c) Variability in the carbon fixation rate and substrate limitation in the aquaria

Most previous work measuring the rates of carbon fixation by intact hydrothermal vent symbioses have been performed on low numbers of individuals [4,5,27,39], and used the resulting collective effect on the chemical composition of the seawater outflow and the total biomass to estimate mass-specific metabolic rates. Here, the use of stable isotopically labelled inorganic carbon in our aquarium seawater allowed us to examine the variability in productivity among individuals in our variation experiment incubations, where we interrogated the mass-specific rates of carbon fixation for between five and 10 individuals per genus. We found that the most productive animals in each aquarium likely accounted for much of the sulfur oxidation occurring in each aquaria. Using the molar ratios of 6.21 and 6.64 for the amount of carbon fixed per sulfide or thiosulfate [50] with an assumed 10% efficiency of energy conservation, we calculated the predicted rate of sulfur oxidation by the most productive animals in each aquarium from their individual rates of carbon fixation. In the sulfide treatments, sulfide oxidation by the two most productive individuals may have accounted for 27% and 51% of the total oxidation in the *Alviniconcha* spp. and *I. nautilei* aquaria, respectively. In the thiosulfate treatments, oxidation by the productive individuals (all other individuals showed no carbon incorporation) may have accounted for 100% and 64% (*Alviniconcha* spp*.* and *I. nautilei*, respectively) of the total oxidation in each aquarium.

The striking disparity in carbon fixation rates among individuals was unlikely due to biological diversity (e.g. differences in symbiont populations). The symbionts of all *I. nautilei* and *B. brevior* exhibited low diversity across all experiments. Though individuals of the *Alviniconcha* spp. in the sulfide treatment of the variation experiment were dominated by either *ɛ*-proteobacterial or *γ*-1 symbionts, rates of incorporation did not appear to be related to symbiont identity (figure 3*a*). Instead, these disparities in carbon incorporation may be due to differences in the availability of resources along the length of the aquaria. We discovered that the individuals closest to the water input incorporated the greatest amount of carbon, and those near the outflow showed no measurable carbon incorporation, indicating that the most productive individuals near the input were depleting some substrate for those downstream. This pattern was clearly observed in the variation experiments (figure 3). Because both sulfur and oxygen were not entirely depleted in the effluent, it is unclear whether these substrates, or any other substrate for that matter, were restricting the productivity of the symbioses. It is also plausible that waste products from the more productive individuals may have inhibited productivity by those located downstream, though it is unclear what those waste products might be (the predominant waste products of chemoautotrophic sulfide oxidation are oxidized sulfur compounds and hydrogen ions) [43,53].

Our results have important implications for our understanding of total productivity of assemblages of these organisms in their habitats, particularly if the observed variability in carbon fixation rates is due to sulfur limitation. Large communities of these symbioses are often found piled around hydrothermal vent orifices, often two to seven animals deep (C. Fisher 2013, personal communication). In these piles, an individual's access to sulfide (or any vent-derived substrate)—as well as its exposure to waste products from other animals—is governed by the confluence of end-member concentration, fluid flow rate and animal/microbial uptake rate. *In situ* populations are likely experiencing gradients in vent-derived geochemistry resulting from the net effect of biotic and abiotic factors. Our data suggest that competition for vent-derived resources, which is tied to spatial position in the fluid flow relative to other individuals, may be significant for these symbioses. Interestingly, these results also provide another perspective from which to view thiosulfate-driven autotrophy in these ecosystems. At the tops of the assemblages, where vent-derived reductants may be depleted by the activity of those below, use of an energy source that is not directly sourced from venting fluid could sustain productivity. Therefore, metabolic flexibility has the potential to relieve competition for vent-derived reductants, both within and among species.

### (d) Excretion of sulfur compounds

Though links between the distribution of particular ELSC symbioses and elevated concentrations of partially oxidized sulfur may indicate a preference for that particular geochemical niche, it is also possible that such correlation may result from excretion of that compound by the associated symbioses. To address sulfur excretion by the ELSC symbioses, we measured the production of partially oxidized sulfur in our incubations. The sulfide treatment in our variation experiment showed that these symbioses have the potential to contribute to the partially oxidized sulfur pools in their environment. During the approximately 350 µM sulfide variation experiment, *I. nautilei* released polysulfides (as previously described in Gartman *et al*. [13]). We quantified mass-specific rates, showing that this excretion stoichiometrically represented up to approximately 50% of the sulfur oxidized in that aquaria. Additionally, both *Alviniconcha* spp. and *B. brevior* excreted thiosulfate, though the mass-specific rate was nine times higher in *Alviniconcha* spp. Because this *Alviniconcha* incubation included individuals hosting either *ɛ*-proteobacterial or *γ*-1 symbionts, the extent to which these phylotypes contributed to the observed excretion remains unclear. Furthermore, as our incubations were performed on intact symbioses, we are unable to discern which partner, host or symbiont, is the source of the excreted sulfur. Many invertebrates, even those without chemoautotrophic symbionts, detoxify sulfide via oxidation to thiosulfate in their mitochondria [49]. Because we did not observe sulfur excretion in the other incubation with a lower sulfide concentration (i.e. the rate experiment with approx. 105 μM sulfide), this could be the case. Additionally, *B. brevior* did not fix carbon when exposed to approximately 350 μM sulfide, though sulfide oxidation was observed. The sulfide concentration in this incubation is likely higher than what *B. brevior* would experience *in situ* [40–42], suggesting the possibility that it was oxidizing sulfide to thiosulfate for detoxification.

POSCs may also be the product of sulfide oxidation by the symbionts of these animals. The symbionts of the vent tubeworm *Riftia pachyptila* produce polysulfides during sulfide oxidation *in vitro* [27], which is thought to be a normal intermediate during the production of sulfur granules as it is with other sulfide oxidizers [54]. Experiments with the sulfur-oxidizing isolate *Thiobacillus thioparus* showed that both thiosulfate and polysulfides can be produced via sulfide oxidation when oxygen is limiting [55]. Though more than 5 μM oxygen was always detected in the effluent of the aquaria (data not shown), respiration by the high biomass in the sulfide variation experiment could have caused low local concentrations in the aquaria, resulting in high sulfide to oxygen ratios. Additionally, host physiology likely mediates the intracellular oxygen concentrations experienced by the symbionts, and it is possible that oxygen was limiting for them despite conditions in the aquaria.

Regardless of the organism of origin, net excretion of partially oxidized sulfur by these symbioses reveals a biological source for these compounds *in situ*. Higher polysulfide concentrations are detected around *I. nautilei* and *Alviniconcha* spp. at the ELSC, whereas higher thiosulfate concentrations are found around *B. brevior* [17]. It was suggested previously that these POSCs result from the abiotic oxidation of sulfide in venting fluid by aqueous iron or rocky substrate, or from biological oxidation by the symbioses. Here, we demonstrate that biological oxidation influences the presence of these sulfur compounds, ultimately affecting the local sulfur regime. As both free-living microbes and vent symbioses can use these compounds for autotrophy, biological sulfur transformations have the potential to affect the distribution and activities of many organisms within these ecosystems.

## 5. Conclusion

The extent to which vent symbioses can use exogenous thiosulfate to drive autotrophy remains unknown, though genomic analyses of the symbionts of vent organisms has revealed that many possess the metabolic pathway for the oxidation of sulfur, including thiosulfate [56–61]. Thiosulfate concentrations at vent habitats have only been extensively documented at the ELSC, but given the abiotic oxidation of sulfide to thiosulfate in the presence of certain metals [62,63], as well as the rapid oxidation of sulfur by many microorganisms, POSCs are likely present in these and other systems [64]. Thiosulfate-fueled autotrophy has potential ecological benefits, particularly for symbioses that cannot be near the high concentrations of reductants in venting fluid, either due to physiological intolerance to high temperatures or toxic vent chemicals or because of competitive exclusion. Additionally, flexible use of multiple reductants may help vent symbioses cope with periods of low exposure to reductants in vent fluid that result from the temporal and spatial inconsistency of these habitats. Though we showed that access to particular sulfur compounds differentially affects the productivity of these symbioses, the ability of all three coexisting genera to fuel autotrophy with both sulfide and thiosulfate implies that metabolic flexibility has important advantages in these ecosystems.

While it has long been expected that vent symbioses can alter the local geochemical regime through the uptake and oxidation of sulfide, the observed excretion of partially oxidized sulfur establishes a new mode of chemical interaction and alteration within their ecosystem. Since the discovery of hydrothermal vents, sulfide has been known to play a fundamental role in structuring and supporting vent assemblages [65]. Here, our data reveal an influence of vent symbioses on sulfur biogeochemical cycling beyond the acquisition and complete oxidation of sulfide. These symbioses may also be influencing the availability of POSCs that are of energetic value to the free-living microbes in these ecosystems. Tubeworm symbioses have previously been described as ecosystem engineers for the role they play in shaping the physical structure of their environment [66]; our data illustrate the extent to which chemoautotrophic symbioses might govern the local sulfur regime as well.

## Supplementary Material

Supplementary Material

## Data accessibility

DNA sequences: GenBank accession KF780847–55. Stable isotopic data: uploaded as electronic supplementary material.
