# Supplementary material for: The uptake and excretion of partially oxidized sulfur expands the repertoire of energy resources metabolized by hydrothermal vent symbioses
Source: Proc Biol Sci. 2015 May 7;282(1806):20142811. doi: 10.1098/rspb.2014.2811 (PMC4426611; doi:10.1098/rspb.2014.2811)
Supplement: Supplementary Material [file rspb20142811supp1.docx]

**Supplementary Methods**

*Incubations with the high-pressure respirometry system (HPRS)*

To measure sulfur oxidation and carbon fixation rates under *in situ*-like conditions, rate experiments and exposure treatments were performed with the HPRS (Fig.1). The HPRS was housed in a temperature-controlled intermodal shipping container maintained at 15-17°C. Surface seawater from the ship’s metal-free seawater systems was filtered to 0.2 μm via inline cartridge filters (Millipore Inc), then pumped into a 40 L polypropylene carboy (Nalgene™). Filtered seawater was then amended with sodium carbonate (Na^13^CO_3_; 99.9% atom percent; Icon Services), to achieve a final ^13^C/^12^C atom percent of ~5%. In addition, sodium nitrate (NaNO_3_) was added to achieve a final concentration of 40 µM, comparable to deep ocean water.

For the thiosulfate experiment and treatment, 300 mM sodium thiosulfate (NaS_2_O_3_) was added to the amended seawater in carboy to achieve a final concentration of 300 μM. This was pumped into an acrylic gas equilibration column [1,2], where it was bubbled with carbon dioxide, oxygen, and nitrogen using mass flow controllers (Sierra Instruments Inc) to achieve concentrations of 4 mM, >300 µM, and 400 µM respectively (Table S2). The pH of the resulting input water was always 6-7. For the hydrogen sulfide experiment and treatment, the conditions were identical except for the absence of the thiosulfate, and the addition of gaseous 5% H_2_S/95% N_2_ gas via a mass flow controller to achieve the target final concentrations (presented above).

In all incubations, the resulting seawater from the equilibration column was then supplied to four high-pressure metering pumps (Lewa GmbH) equipped with titanium wetted parts. The pumps generated ~25 mPa and delivered fluid into the three or four titanium high-pressure aquaria at a rate of 10-16 ml min^-1^. Pressure was maintained via 316 stainless steel backpressure valves (StraVal Inc). The aquaria effluents and/or equilibration column seawater (hereafter ‘input water’) were directed toward an electronic, multi-position stream-selection valve (Valco Instruments Co. Inc.) that systematically sent each stream to analysis by a voltammetric microelectrode (see below), to collection for analyses on shore, or to waste.

*Cyclic voltammetric calibration and limits of detection*

The detection limits for the voltammetric microelectrodes were 30 μM thiosulfate and 0.2 μM sulfide and polysulfide. The electrodes were calibrated for measurement of sulfide concentrations with measurements made via discrete water samples from either the input water (exposure treatment) or the control vessel effluent (rate experiment). The electrodes were calibrated for measurement of thiosulfate concentration based on the 300 μM concentration in the input water.

To determine total sulfide concentrations, 10 ml of input or control effluent water was collected every 4 hours, and preserved with 1 M zinc acetate and stored at -20°C until analysis. Sulfide concentrations were determined via a colorimetric assay ([3-5] on a Spectramax Plus 384 absorbance microplate reader (Molecular Devices, LLC).

*DIC isotopic composition*

To quantify the amount of isotopic tracer Na^13^CO_3_ available during each experiment or treatment, input water was sampled 2-3 times over the course of each. Approximately 10 ml of input water taken directly from the gas equilibration column was filtered (0.2 μm) to remove particulate carbon. Dissolved inorganic carbon (DIC) in the samples was base-trapped with a solution of sodium hydroxide so that the final pH was >11 and stored frozen at -20°C in gas-tight, glass Hungate tubes until analysis. The atom percent of the DIC was measured at the Yale Institute of Biospheric Studies’ Earth System Center for Stable Isotopic Studies, where 1 ml of thawed sample was injected into pre-flushed 12 ml exetainers containing H_3_PO_4_ to evolve DIC as CO_2_ for analysis via a ThermoFinnigan Delta^PLUS^ Advantage mass spectrometer (Thermo Scientific) coupled to a Costech ECS 4010 EA elemental analyzer (Costech Analytical Technologies).

*Symbiont identities*

DNA was back-extracted from the Trizol™-preserved tissue samples following RNA extraction via the manufacturer’s protocol. Specifically, DNA was back-extracted from the resulting interphase with a buffer consisting of 4 M guanidine thiocyanate, 50 mM sodium citrate and 1 M Tris (free base). The DNA was then extracted again with chloroform:isoamyl alcohol and precipitated with isopropanol. The resulting DNA pellets were washed twice with 75% ethanol and air-dried. DNA pellets were resuspended in 8 mM sodium hydroxide, adjusted to pH 7-8 with 0.1 M HEPES and amended with 1 mM EDTA.

Symbiont 16S rRNA genes were amplified from diluted *I. nautilei* and *B. brevior* DNA extracts using the universal bacterial primers 27F and 1492R [3,6,7]. PCR reactions were performed with Crimson Taq DNA polymerase (New England Biolabs, Inc.) for 2 min at 95 °C, 30 cycles of 30 s at 95°C, 30 s at 55°C, 90 s at 68°C, followed by 5 min at 72°C. PCR products were subjected to electrophoresis on a 1.2% (wt/vol) agarose gel stained with SYBR Safe (Invitrogen, Inc.) to check the quantity and the quality of the products using a U:Genius UV transilluminator (Syngene, Inc.). PCR products were cleaned with ExoSAP-IT (Affymetrix, Inc.), then bidirectionally sequenced. Quality assessment of the sequences and assembly of forward and reverse reads were performed in Geneious v6.1.6 (BioMatters, Inc.). Sequences were aligned with other symbiont and free-living Proteobacterial sequences using the SILVA Incremental Aligner v1.2.11 [8,9]. A Bayesian inference phylogeny was produced with MrBayes

[10] implementing the GTR+I+G model of substitution. Three replicate runs of 5 x 10^7^ generations were performed with sampling every 10^3^ generations and burn-in of 12,500 samples. Quantitative and qualitative diagnostics were performed for each of the three runs using the Coda package in R [1], the three replicate runs were combined, and a 50% majority rule consensus tree was created in MrBayes.

Because *Alviniconcha* from the ELSC are known to host an assemblage of phylogenetically distinct symbionts [3], direct amplification and sequencing of symbiont 16S rRNA genes was not performed. Instead, the symbiont populations associated with the experimental *Alviniconcha* were assessed as in Beinart *et al*. (2012), with three quantitative PCR assays that are specific to their symbiont phylotypes. Briefly, we estimated the proportion of each symbiont phylotype in the diluted *Alviniconcha* gill DNA extracts by applying all the assays to 2 μl of each sample (in duplicate), which were compared against duplicate standard curves and no-template controls. A standard curve for each assay was constructed from linearized plasmid containing a representative 16S rRNA gene from the three symbiont phylotypes, diluted so that 10^1^ to 10^7^ gene copies were added per reaction.

*Carbon stable isotopic analyses*

Approximately 300 mg symbiont-containing gill and symbiont-free foot tissue were subsampled while frozen for carbon isotopic analysis. Samples were lyophilized for 24 h and then were acidiﬁed with 0.1 N HCl to remove any unincorporated Na^13^CO_3_ contamination. The samples were subsequently dried for 24–48 h at 50–60°C, weighed to determine the dry weight, homogenized to a ﬁne powder, and ~1 mg sealed within tin capsules. The carbon isotopic composition and percent carbon content were determined for foot tissue samples at Washington State University by combustion in an elemental analyzer (Eurovector, Inc.) and separating the evolved CO_2_ by gas chromatography before introduction to a Isoprime™ isotope ratio mass spectrometer (Micromass Inc). Gill tissue samples were assayed at the Yale Institute of Biospheric Studies’ Earth System Center for Stable Isotopic Studies using a ThermoFinnigan Delta^PLUS^ Advantage mass spectrometer (Thermo Scientific) coupled to a Costech ECS 4010 EA elemental analyzer (Costech Analytical Technologies). Measurements of isotopic composition are expressed as the atomic percent (%A = [^13^C/(^13^C+^12^C)] x 100%) and carbon contents expressed as a percentage of dry weight (%C) (Table S1).

The average isotopic composition of the foot tissue of each genus from all experiments was comparable to the natural isotopic composition of these animals (Table S3), indicating that ^13^C incorporation into foot tissue or contamination of the samples did not occur. Foot tissue was not sampled at the conclusion of the sulfide exposure treatment, so the average A%_f_ from the other experiments was used to calculate rates for the individuals in this experiment (averages 1.076%, 1.075%, and 1.075% for *Alviniconcha*, *I. nautilei* and *B. brevior*, respectively). For the *Alviniconcha* individuals in the sulfide exposure treatment that hosted ε-proteobacterial symbionts, the A%_f_ average from the previous experiments could not be used since *Alviniconcha* from the other experiments hosted γ-proteobacterial symbionts. For these individuals, the previously published average A%_g_ (1.093%; [3]) for ε-proteobacteria-hosting individuals was used since gill tissue is typically a reasonable approximation of the A%_f_ in *Alviniconcha* [8].

**Supplementary Tables**

**Table S1:** Experimental and stable isotopic data used to calculate mass-specific carbon incorporation rates.

| **Experiment** | **Genus** | **Individual** | **gill weight (g)** | **time (hours)** | **%Dry Weight (g)** | **mass wet tissue (mg)** | **%C** | **δ13C gill** | **%A gill** | **%A foot** | **%A DIC** |
| --- | --- | --- | --- | --- | --- | --- | --- | --- | --- | --- | --- |
| Sulfide variation | *Alviniconcha* spp. | 1 | 3.65 | 35.83 | 0.21 | 1.00 | 31.87 | -9.06 | 1.09570 | 1.09292 | 6.45170 |
| Sulfide variation | *Alviniconcha* spp. | 2 | 4.38 | 35.83 | 0.22 | 1.00 | 51.72 | -27.41 | 1.07562 | 1.07611 | 6.45170 |
| Sulfide variation | *Alviniconcha* spp. | 3 | 2.84 | 35.83 | 0.19 | 1.00 | 69.81 | -23.05 | 1.08040 | 1.07611 | 6.45170 |
| Sulfide variation | *Alviniconcha* spp. | 4 | 3.46 | 35.83 | 0.19 | 1.00 | 61.49 | -25.67 | 1.07753 | 1.07611 | 6.45170 |
| Sulfide variation | *Alviniconcha* spp. | 5 | 6.98 | 35.83 | 0.19 | 1.00 | 47.47 | -22.45 | 1.08105 | 1.07611 | 6.45170 |
| Sulfide variation | *Alviniconcha* spp. | 6 | 3.11 | 35.83 | 0.19 | 1.00 | 49.84 | -9.55 | 1.09516 | 1.09292 | 6.45170 |
| Sulfide variation | *Alviniconcha* spp. | 7 | 2.76 | 35.83 | 0.19 | 1.00 | 56.63 | -0.44 | 1.10512 | 1.09292 | 6.45170 |
| Sulfide variation | *Alviniconcha* spp. | 8 | 3.45 | 35.83 | 0.19 | 1.00 | 48.68 | 0.14 | 1.10575 | 1.09292 | 6.45170 |
| Sulfide variation | *Alviniconcha* spp. | 9 | 3.13 | 35.83 | 0.21 | 1.00 | 62.08 | 80.78 | 1.19384 | 1.09292 | 6.45170 |
| Sulfide variation | *Alviniconcha* spp. | 10 | 3.08 | 35.83 | 0.19 | 1.00 | 67.33 | 143.52 | 1.26228 | 1.09292 | 6.45170 |
| Sulfide variation | *I. nautilei* | 1 | 9.73 | 37.70 | 0.49 | 1.00 | 40.58 | -32.77 | 1.06975 | 1.07503 | 6.45170 |
| Sulfide variation | *I. nautilei* | 2 | 4.31 | 37.70 | 0.19 | 1.00 | 38.25 | -33.41 | 1.06906 | 1.07503 | 6.45170 |
| Sulfide variation | *I. nautilei* | 3 | 6.08 | 37.70 | 0.25 | 0.97 | 48.68 | -29.74 | 1.07308 | 1.07503 | 6.45170 |
| Sulfide variation | *I. nautilei* | 4 | 7.05 | 37.70 | 0.22 | 1.00 | 60.07 | 1.41 | 1.10714 | 1.07503 | 6.45170 |
| Sulfide variation | *I. nautilei* | 5 | 6.91 | 37.70 | 0.20 | 0.98 | 46.89 | 217.29 | 1.34261 | 1.07503 | 6.45170 |
| Sulfide variation | *B. brevior* | 1 | 7.88 | 36.98 | 0.14 | 1.00 | 10.57 | -32.37 | 1.07019 | 1.07482 | 6.45170 |
| Sulfide variation | *B. brevior* | 2 | 8.47 | 36.98 | 0.12 | 1.00 | 52.35 | -32.93 | 1.06958 | 1.07482 | 6.45170 |
| Sulfide variation | *B. brevior* | 3 | 8.23 | 36.98 | 0.14 | 1.00 | 49.06 | -33.89 | 1.06853 | 1.07482 | 6.45170 |
| Sulfide variation | *B. brevior* | 4 | 0.96 | 36.98 | 0.14 | 1.00 | 68.00 | -33.25 | 1.06923 | 1.07482 | 6.45170 |
| Sulfide variation | *B. brevior* | 5 | 6.72 | 36.98 | 0.15 | 1.00 | 70.17 | -32.98 | 1.06953 | 1.07482 | 6.45170 |
| Sulfide variation | *B. brevior* | 6 | 7.63 | 36.98 | 0.14 | 1.00 | 38.26 | -31.35 | 1.07131 | 1.07482 | 6.45170 |
| **Experiment** | **Genus** | **Individual** | **gill weight (g)** | **time (hours)** | **%Dry Weight (g)** | **mass wet tissue (mg)** | **%C** | **δ13C gill** | **%A gill** | **%A foot** | **%A DIC** |
| Thiosulfate variation | *Alviniconcha* spp. | 1 | 3.45 | 36.07 | 0.21 | 1.02 | 44.05 | -29.73 | 1.07308 | 1.07503 | 3.37567 |
| Thiosulfate variation | *Alviniconcha* spp. | 2 | 6.78 | 36.07 | 0.18 | 0.96 | 47.55 | 65.12 | 1.17675 | 1.07566 | 3.37567 |
| Thiosulfate variation | *Alviniconcha* spp. | 3 | 3.12 | 36.07 | 0.20 | 1.00 | 46.85 | -28.39 | 1.07455 | 1.07761 | 3.37567 |
| Thiosulfate variation | *Alviniconcha* spp. | 4 | 6.45 | 36.07 | 0.20 | 1.05 | 45.84 | -24.28 | 1.07905 | 1.07566 | 3.37567 |
| Thiosulfate variation | *Alviniconcha* spp. | 5 | 2.32 | 36.07 | 0.20 | 0.98 | 50.18 | 14.23 | 1.12116 | 1.07782 | 3.37567 |
| Thiosulfate variation | *Alviniconcha* spp. | 6 | 6.61 | 36.07 | 0.17 | 0.95 | 51.21 | 120.02 | 1.23665 | 1.07826 | 3.37567 |
| Thiosulfate variation | *Alviniconcha* spp. | 7 | 3.07 | 36.07 | 0.19 | 1.06 | 48.30 | -29.17 | 1.07369 | 1.07552 | 3.37567 |
| Thiosulfate variation | *Alviniconcha* spp. | 8 | 7.47 | 36.07 | 0.22 | 1.04 | 50.36 | -30.56 | 1.07217 | 1.07937 | 3.37567 |
| Thiosulfate variation | *I. nautilei* | 1 | 7.52 | 37.22 | 0.22 | 1.03 | 30.01 | -33.56 | 1.06889 | 1.07503 | 3.37567 |
| Thiosulfate variation | *I. nautilei* | 2 | 4.28 | 37.22 | 0.22 | 1.05 | 47.26 | -33.05 | 1.06946 | 1.07480 | 3.37567 |
| Thiosulfate variation | *I. nautilei* | 3 | 6.42 | 37.22 | 0.19 | 1.05 | 52.17 | -34.35 | 1.06803 | 1.07485 | 3.37567 |
| Thiosulfate variation | *I. nautilei* | 4 | 4.80 | 37.22 | 0.17 | 1.00 | 52.33 | -33.38 | 1.06909 | 1.07488 | 3.37567 |
| Thiosulfate variation | *I. nautilei* | 5 | 4.31 | 37.22 | 0.19 | 1.05 | 51.37 | -30.85 | 1.07186 | 1.07477 | 3.37567 |
| Thiosulfate variation | *I. nautilei* | 8 | 3.67 | 37.22 | 0.19 | 0.99 | 52.06 | 49.10 | 1.15926 | 1.07543 | 3.37567 |
| Thiosulfate variation | *I. nautilei* | 9 | 6.00 | 37.22 | 0.18 | 0.96 | 53.11 | 36.10 | 1.14506 | 1.07401 | 3.37567 |
| Thiosulfate variation | *I. nautilei* | 10 | 5.72 | 37.22 | 0.22 | 0.98 | 49.28 | 23.71 | 1.13151 | 1.07590 | 3.37567 |
| Thiosulfate variation | *B. brevior* | 1 | 8.77 | 38.37 | 0.13 | 1.00 | 48.08 | -32.67 | 1.06987 | 1.07637 | 3.37567 |
| Thiosulfate variation | *B. brevior* | 2 | 8.87 | 38.37 | 0.10 | 0.98 | 46.77 | 16.67 | 1.12382 | 1.07807 | 3.37567 |
| Thiosulfate variation | *B. brevior* | 3 | 6.44 | 38.37 | 0.13 | 1.04 | 47.34 | 16.74 | 1.12390 | 1.08061 | 3.37567 |
| Thiosulfate variation | *B. brevior* | 4 | 12.41 | 38.37 | 0.11 | 0.97 | 44.62 | -32.73 | 1.06980 | 1.07389 | 3.37567 |
| Thiosulfate variation | *B. brevior* | 5 | 9.02 | 38.37 | 0.11 | 0.97 | 46.41 | -33.11 | 1.06938 | 1.07359 | 3.37567 |
| Thiosulfate variation | *B. brevior* | 6 | 8.55 | 38.37 | 0.08 | 1.04 | 48.39 | -32.90 | 1.06961 | 1.07223 | 3.37567 |
|  |  |  |  |  |  |  |  |  |  |  |  |
|  |  |  |  |  |  |  |  |  |  |  |  |
|  |  |  |  |  |  |  |  |  |  |  |  |
| **Experiment** | **Genus** | **Individual** | **gill weight (g)** | **time (hours)** | **%Dry Weight (g)** | **mass wet tissue (mg)** | **%C** | **δ13C gill** | **%A gill** | **%A foot** | **%A DIC** |
| Thiosulfate rate | *Alviniconcha* spp. | 1 | 7.89 | 28.52 | 0.23 | 1.10 | 31.01 | 7.24 | 1.11351 | 1.07535 | 4.82714 |
| Thiosulfate rate | *Alviniconcha* spp. | 2 | 9.43 | 28.52 | 0.20 | 1.00 | 11.71 | -11.27 | 1.09327 | 1.07495 | 4.82714 |
| Thiosulfate rate | *Alviniconcha* spp. | 3 | 4.11 | 28.52 | 0.19 | 1.00 | 37.70 | -20.39 | 1.08331 | 1.07542 | 4.82714 |
| Thiosulfate rate | *I. nautilei* | 1 | 4.51 | 29.52 | 0.24 | 1.00 | 51.48 | 15.16 | 1.12218 | 1.07271 | 4.82714 |
| Thiosulfate rate | *I. nautilei* | 3 | 4.56 | 29.52 | 0.25 | 1.00 | 50.76 | 46.02 | 1.15589 | 1.07686 | 4.82714 |
| Thiosulfate rate | *I. nautilei* | 4 | 3.46 | 29.52 | 0.27 | 1.00 | 58.09 | -14.63 | 1.08960 | 1.07328 | 4.82714 |
| Thiosulfate rate | *B. brevior* | 1 | 2.51 | 30.52 | 0.11 | 1.00 | 57.90 | -30.71 | 1.07201 | 1.07207 | 4.82714 |
| Thiosulfate rate | *B. brevior* | 2 | 2.91 | 30.52 | 0.11 | 0.80 | 30.21 | -28.75 | 1.07415 | 1.07368 | 4.82714 |
| Thiosulfate rate | *B. brevior* | 3 | 8.04 | 30.52 | 0.11 | 1.00 | 46.53 | -31.23 | 1.07144 | 1.07330 | 4.82714 |
| Sulfur-free | *Alviniconcha* spp. | 1 | 3.17 | 21.15 | 0.21 | 1.04 | 43.19 | -30.01 | 1.07277 | 1.07498 | 5.08179 |
| Sulfur-free | *Alviniconcha* spp. | 2 | 3.77 | 21.15 | 0.22 | 0.99 | 46.75 | -29.79 | 1.07302 | 1.07462 | 5.08179 |
| Sulfur-free | *Alviniconcha* spp. | 3 | 4.11 | 21.15 | 0.21 | 1.03 | 47.06 | -30.01 | 1.07278 | 1.07523 | 5.08179 |
| Sulfur-free | *Alviniconcha* spp. | 4 | 2.40 | 21.15 | 0.16 | 0.95 | 44.79 | -27.90 | 1.07508 | 1.07668 | 5.08179 |
| Sulfur-free | *Alviniconcha* spp. | 5 | 2.44 | 21.15 | 0.16 | 1.05 | 44.76 | -27.22 | 1.07583 | 1.07637 | 5.08179 |
| Sulfur-free | *I. nautilei* | 1 | 3.03 | 21.92 | 0.18 | 0.97 | 49.85 | -31.40 | 1.07125 | 1.07549 | 5.08179 |
| Sulfur-free | *I. nautilei* | 2 | 5.37 | 21.92 | 0.19 | 1.04 | 45.76 | -30.82 | 1.07189 | 1.07512 | 5.08179 |
| Sulfur-free | *I. nautilei* | 3 | 2.70 | 21.92 | 0.17 | 1.00 | 50.52 | -31.16 | 1.07152 | 1.07536 | 5.08179 |
| Sulfur-free | *I. nautilei* | 4 | 3.33 | 21.92 | 0.14 | 1.01 | 47.19 | -30.73 | 1.07199 | 1.07578 | 5.08179 |
| Sulfur-free | *I. nautilei* | 5 | 4.41 | 21.92 | 0.23 | 1.03 | 50.09 | -31.19 | 1.07149 | 1.07503 | 5.08179 |
| Sulfur-free | *B. brevior* | 1 | 4.32 | 22.60 | 0.34 | 1.05 | 43.34 | -30.56 | 1.07218 | 1.07436 | 5.08179 |
| Sulfur-free | *B. brevior* | 2 | 4.62 | 22.60 | 0.46 | 1.02 | 43.60 | -30.49 | 1.07225 | 1.07395 | 5.08179 |
| Sulfur-free | *B. brevior* | 3 | 5.08 | 22.60 | 0.17 | 1.03 | 46.98 | -27.39 | 1.07565 | 1.07461 | 5.08179 |
| Sulfur-free | *B. brevior* | 4 | 4.76 | 22.60 | 0.09 | 1.05 | 46.23 | -24.69 | 1.07860 | 1.07392 | 5.08179 |
|  |  |  |  |  |  |  |  |  |  |  |  |
|  |  |  |  |  |  |  |  |  |  |  |  |
| **Experiment** | **Genus** | **Individual** | **gill weight (g)** | **time (hours)** | **%Dry Weight (g)** | **mass wet tissue (mg)** | **%C** | **δ13C gill** | **%A gill** | **%A foot** | **%A DIC** |
| Sulfide rate | *Alviniconcha* spp. | 1 | 4.19 | 38.87 | 0.19 | 0.94 | 45.09 | -26.13 | 1.07672 | 1.07657 | 5.43724 |
| Sulfide rate | *Alviniconcha* spp. | 2 | 3.25 | 38.87 | 0.20 | 1 | 41.60 | -3.77 | 1.10143 | 1.07615 | 5.43724 |
| Sulfide rate | *Alviniconcha* spp. | 3 | 6.72 | 38.87 | 0.21 | 0.95 | 48.19 | -21.29 | 1.08206 | 1.07655 | 5.43724 |
| Sulfide rate | *Alviniconcha* spp. | 4 | 5.61 | 38.87 | 0.08 | 0.94 | 45.98 | -27.21 | 1.07552 | 1.07459 | 5.43724 |
| Sulfide rate | *Alviniconcha* spp. | 5 | 2.78 | 38.87 | 0.20 | 1.02 | 47.40 | -14.04 | 1.09008 | 1.07588 | 5.43724 |
| Sulfide rate | *I. nautilei* | 3 | 3.73 | 39.68 | 0.11 | 1 | 45.56 | 2.85 | 1.10875 | 1.07564 | 5.43724 |
| Sulfide rate | *I. nautilei* | 4 | 4.11 | 39.68 | 0.13 | 0.98 | 52.49 | -28.69 | 1.07388 | 1.07514 | 5.43724 |
| Sulfide rate | *I. nautilei* | 5 | 4.24 | 39.68 | 0.23 | 1.05 | 49.53 | -32.00 | 1.07022 | 1.07469 | 5.43724 |
| Sulfide rate | *B. brevior* | 1 | 5.28 | 40.35 | 0.12 | 1.01 | 48.72 | -29.06 | 1.07347 | 1.07522 | 5.43724 |
| Sulfide rate | *B. brevior* | 2 | 6.70 | 40.35 | 0.16 | 1.01 | 43.94 | 28.89 | 1.13718 | 1.07471 | 5.43724 |
| Sulfide rate | *B. brevior* | 3 | 3.11 | 40.35 | 0.31 | 1.02 | 45.72 | -27.74 | 1.07493 | 1.07636 | 5.43724 |
| Sulfide rate | *B. brevior* | 4 | 3.26 | 40.35 | 0.12 | 0.98 | 45.46 | 37.30 | 1.14684 | 1.07503 | 5.43724 |
|  |  |  |  |  |  |  |  |  |  |  |  |
|  |  |  |  |  |  |  |  |  |  |  |  |

**Table S2:** Proportions of symbiont phylotypes associating with *Alviniconcha* as assessed via quantitative PCR.

| Experiment/Treatment | Individual | %γ-1 | %γ-Lau | %ε |
| --- | --- | --- | --- | --- |
| Sulfur-free | 1 | 100 | 0 | 0 |
|  | 2 | 99 | 1 | 0 |
|  | 3 | 100 | 0 | 0 |
|  | 4 | 3 | 97 | 0 |
|  | 5 | 3 | 96 | 0 |
| Sulfide | 1 | 100 | 0 | 0 |
|  | 2 | 100 | 0 | 0 |
|  | 3 | 93 | 7 | 1 |
|  | 4 | 99 | 1 | 0 |
|  | 5 | 96 | 0 | 4 |
| Thiosulfate | 1 | 100 | 0 | 0 |
|  | 2 | 99 | 0 | 1 |
|  | 3 | 100 | 0 | 0 |
| Sulfide treatment | 1 | 0 | 0 | 100 |
|  | 2 | 99 | 0 | 1 |
|  | 3 | 99 | 0 | 1 |
|  | 4 | 100 | 0 | 0 |
|  | 5 | 100 | 0 | 0 |
|  | 6 | 0 | 0 | 100 |
|  | 7 | 0 | 0 | 100 |
|  | 8 | 0 | 0 | 100 |
|  | 9 | 0 | 0 | 100 |
|  | 10 | 0 | 0 | 100 |
| Thiosulfate treatment | 1 | 100 | 0 | 0 |
|  | 2 | 100 | 0 | 0 |
|  | 3 | 100 | 0 | 0 |
|  | 4 | 100 | 0 | 0 |
|  | 5 | 99 | 0 | 1 |
|  | 6 | 100 | 0 | 0 |
|  | 7 | 100 | 0 | 0 |
|  | 8 | 100 | 0 | 0 |

**Table S2**: Input water conditions for all experiments and treatments. Mean (min, max) sulfide concentrations as determined via a colorimetric assay as applied to discrete water samples of input water; partial pressure of O_2_ and calculated concentration of O_2_ in input water; mean (min, max); and atom percent of ^13^C in dissolved inorganic carbon (DIC).

| Incubation | Mean [sulfide] (min, max) (μM) | pO_2_ (%) | [O_2_] (μM)* | Mean A% DIC (min, max) |
| --- | --- | --- | --- | --- |
| Sulfur-free | NA | 50 | 562 | 5.08 (4.22, 5.59) |
| Sulfide | 105 (57, 137) | 27.5 | 310 | 5.44 (5.25, 5.75) |
| Thiosulfate | 0 (0,0) | 54.8 | 618 | 4.83 (3.70, 5.63) |
| Sulfide treatment | 388 (338,459) | 54.5 | 615 | 6.45 (6.28, 6.62) |
| Thiosulfate treatment | 0 (0,0) | 52.3 | 590 | 3.38 (2.89, 3.74) |
| *Concentration of O_2_ calculated based on concentration of 100% pO_2_ in seawater at 20°C, 35 ppt [4,5,9] | | | | |

**Table S3:** The average (± S.D.) of the stable isotopic composition of experimental foot and natural tissue expressed as δ^13^C (‰).

|  | Experimental Foot | Natural |
| --- | --- | --- |
| *Alviniconcha* | -27.0 ± 1.16 | -27.6 ± 2.30^a^ |
| *I. nautilei* | -28.0 ± 0.95 | -28.5^b^ |
| *B. brevior* | -28.1 ± 1.93 | -30.6 ± 2.52^c^ |

^a^ gill tissue values, Lau Basin, from Beinart et al. [3]

^b^ gill tissue value, Lau Basin from Suzuki et al. [8]

^c^ foot tissue values, North Fiji Basin from Dubilier et al. [11]

**Supplementary Figures**

**Fig.S1**: Schematic of the high-pressure respirometry system (HPRS). Filtered seawater is amended with chemicals to mimic *in situ* conditions, and then pumped through three titanium aquaria containing the symbiotic molluscs, and in some cases, through an additional, empty control aquaria. These aquaria are held at ~25 mPa with back-pressure valves. The input water and/or the aquaria effluent are directed, via a stream-selection valve, to an in-line voltammetric microelectrode system that measures the concentrations of sulfur compounds (modified from Nyholm et al.

[6])

**
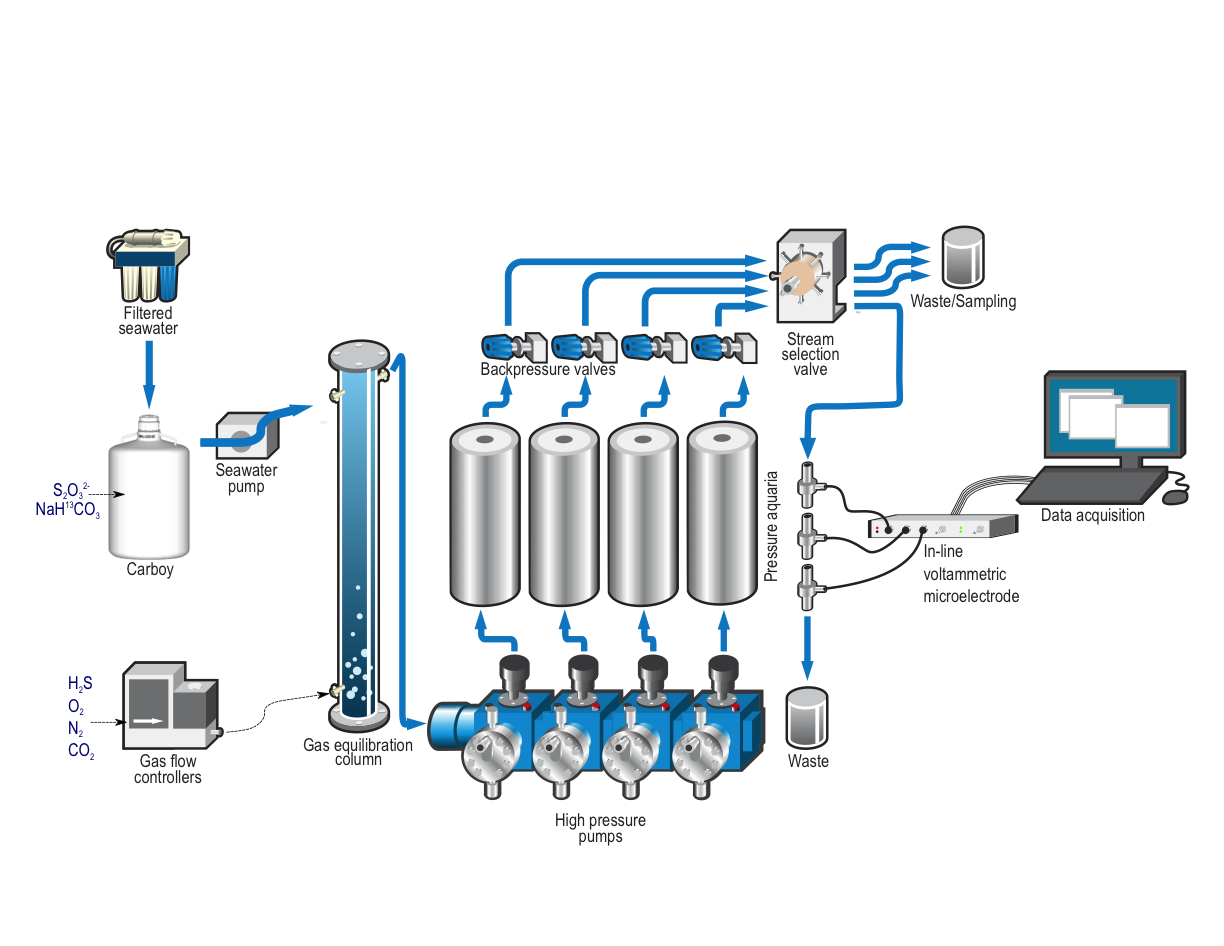
**

**Fig.S2:** Linear regression of gill weight to body weight for each of the three mollusc genera.

**Fig.S3:** Bayesian inference phylogeny of γ-proteobacterial 16S rRNA gene sequences with β-proteobacterial outgroup. The symbionts of B. brevior and I. nautilei from all incubations are shown in bold with the number of individuals yielding that sequence indicated in brackets. Accession numbers are shown in parentheses. Posterior probabilities are indicated above the nodes if >0.7.

**Supplementary References**

1. Plummer, M., Best, N., Cowles, K. & Vines, K. 2006 CODA: convergence diagnosis and output analysis for MCMC. *R News* **6**, 7–11.

2. Girguis, P. R., Lee, R. W., Desaulniers, N., Childress, J. J., Pospesel, M., Felbeck, H. & Zal, F. 2000 Fate of nitrate acquired by the tubeworm *Riftia pachyptila*. *Appl. Environ. Microbiol.* **66**, 2783–2790.

3. Beinart, R. A. et al. 2012 Evidence for the role of endosymbionts in regional-scale habitat partitioning by hydrothermal vent symbioses. *Proceedings of the National Academy of Sciences* **109**, 19053–19054.

4. Weiss, R. F. 1970 The solubility of nitrogen, oxygen and argon in water and seawater. *Deep Sea Research and Oceanographic Abstracts*

5. Cline, J. D. 1969 Spectrophotometric determination of hydrogen sulfide in natural waters. *Limnol. Oceanogr.* **14**, 454–458.

6. Nyholm, S. V., Robidart, J. & Girguis, P. R. 2008 Coupling metabolite flux to transcriptomics: Insights into the molecular mechanisms underlying primary productivity by the hydrothermal vent tubeworm Ridgeia piscesae. *Biol. Bull.* **214**, 255–265.

7. Lane, D. J. 1991 *16S/23S rRNA sequencing*. New York: J. Wiley and Sons.

8. Suzuki, Y. et al. 2006 Host-symbiont relationships in hydrothermal vent gastropods of the genus *Alviniconcha* from the Southwest Pacific. *Appl. Environ. Microbiol.* **72**, 1388–1393.

9. Pruesse, E., Peplies, J. & Glöckner, F. O. 2012 SINA: accurate high-throughput multiple sequence alignment of ribosomal RNA genes. *Bioinformatics* **28**, 1823-1829.

10. Huelsenbeck, J. P. & Ronquist, F. R. 2001 MRBAYES: Bayesian inference of phylogeny. *Bioinformatics* **17**, 754–755.

11. Dubilier, N., Windoffer, R. & Giere, O. 1998 Ultrastructure and stable carbon isotope composition of the hydrothermal vent mussels *Bathymodiolus brevior* and B. sp. affinis *brevior* from the North Fiji Basin, western Pacific. *Mar. Ecol. Prog. Ser.* **165**, 187–193.
